# Dual-Arm Robot Trajectory Planning Based on Deep Reinforcement Learning under Complex Environment

**DOI:** 10.3390/mi13040564

**Published:** 2022-03-31

**Authors:** Wanxing Tang, Chuang Cheng, Haiping Ai, Li Chen

**Affiliations:** 1 School of Energy and Mechanical Engineering, Jiangxi University of Science and Technology, Nanchang 330013, China; n190220054@fzu.edu.cn; 2 College of Mechanical Engineering, Fuzhou University, Fuzhou 350002, China; chnle@fzu.edu.cn; 3 College of Intelligence Science and Technology, National University of Defense Technology, Changsha 410073, China; chengchuang@nudt.edu.cn

**Keywords:** dual-arm robot, deep reinforcement learning, trajectory planning, complex environment, reward

## Abstract

In this article, the trajectory planning of the two manipulators of the dual-arm robot is studied to approach the patient in a complex environment with deep reinforcement learning algorithms. The shape of the human body and bed is complex which may lead to the collision between the human and the robot. Because the sparse reward the robot obtains from the environment may not support the robot to accomplish the task, a neural network is trained to control the manipulators of the robot to prepare to hold the patient up by using a proximal policy optimization algorithm with a continuous reward function. Firstly, considering the realistic scene, the 3D simulation environment is built to conduct the research. Secondly, inspired by the idea of the artificial potential field, a new reward and punishment function was proposed to help the robot obtain enough rewards to explore the environment. The function is consisting of four parts which include the reward guidance function, collision detection, obstacle avoidance function, and time function. Where the reward guidance function is used to guide the robot to approach the targets to hold the patient, the collision detection and obstacle avoidance function are complementary to each other and are used to avoid obstacles, and the time function is used to reduce the number of training episode. Finally, after the robot is trained to reach the targets, the training results are analyzed. Compared with the DDPG algorithm, the PPO algorithm reduces about 4 million steps for training to converge. Moreover, compared with the other reward and punishment functions, the function used in this paper will obtain many more rewards at the same training time. Apart from that, it will take much less time to converge, and the episode length will be shorter; so, the advantage of the algorithm used in this paper is verified.

## 1. Introduction

The arrival of an aging society in the world has increased the social proportion of the elderly population. The care of the elderly has become one of the important concerns of medical care. Recently, COVID-19 has also greatly increased the burden on healthcare. With the progress and development of science and technology, medical robots also began to enter the field of biomedicine. If robots can replace humans to care for patients or the elderly, the burden on society will be greatly reduced [1]. While for the paralytic or seriously injured patient lying on the bed, turning over, or getting up may be a challenging job, it would help a lot if there is a service robot assisting him. Moreover, the interaction between the service robot and the elderly or patients is mainly completed by the robotic arm. Therefore, it is of interest to take on the research on the motion and trajectory planning of the robotic arm.

There are several related works about reinforcement learning and trajectory planning. The emergence of deep reinforcement learning (DRL) solved some of the problems in traditional path planning algorithms, such as A* [2], with hard to construct cost function, artificial potential field (APF) method [3], which is limited by the problem of the local optimum, fast-expanding random tree (FERT) method [4] which is arduous to obtain for an ideal movement trajectory in a narrow area, and so on. DRL does not necessarily rely on data models, and it only requires setting planning goals; then, the robot itself will interact with the environment. Throughout the process, DRL would help avoid obstacles and use path planning to maximize the reward to find an optimal path for a robot [5]. Joshi et al. [6] used imitation learning to study human dressing assistance, but they did not consider obstacle avoidance. To solve this problem, a control algorithm for the whole-body obstacles avoidance of anthropomorphic robots is proposed by Sangiovanni et al. [7]. Wong et al. [8], based on the soft actor–critic (SAC) algorithm, trained the neural network of the robot’s left and right arms agents using dual agent training, distributed training structure, and a progressive training environment. However, the research mentioned above has not involved the interaction between the human and the robot, which is necessary for the robot to explore the environment fully to achieve better performance.

Traditional reinforcement learning (RL) deals with dynamic planning in the state of limited space. Li et al. [9] proposed an integral RL method to calculate the linear quadratic regulation (LQR) to reduce the motion tracking error of the manipulator. Perrusquía et al. [10] used RL to learn the required force when using impedance control to control the force and position of the robot and then generated the required position through proportional-integral admittance control. Ai et al. [11,12] used reinforcement learning to optimize their control effect when studying space robots catching satellites. However, in the references [9,10,11,12], environment models are still indispensable to obtaining the optimal control strategy, and the reliance on environment models remains a problem unsolved. On the contrary, DRL, as a combination of the perception ability of deep learning and the decision-making ability of RL, can handle more complex continuous scenarios with larger action and sample space compared with RL, and it makes the robot interact with the environment directly and master the operation skills. Li et al. [13] designed a DRL-based strategy search method to realize the point-to-point automatic learning of the robotic arm and used a convolutional neural network to maintain the robustness of the robotic arm. Li et al. [14] proposed a method of processing multimodal information with a deep deterministic policy gradient (DDPG) for an assembly robot so that the robot can complete the assembly task without position constraints. However, references [13,14] concerned only the control methods for single-arm robots. Tang et al. [15] compared the control effect of the rapid search random tree (RRT) algorithm and the DDPG algorithm on the coordinated motion planning of space robots, and the results showed that the DDPG algorithm is working with higher efficiency. Beltran-Hernandez et al. [16] proposed a force control framework based on reinforcement learning for the control of rigid robot manipulators, which combined traditional force control methods with the SAC algorithm and thus avoided damage to the environment. Shahid et al. [17] used the proximal policy optimization (PPO) algorithm to study a robot grasping task and designed a rewards and punishments function (RPF) with intensive rewards, but the RPF and requirements of the task are relatively simple. As for the obstacle avoidance of the robotic arm in the process of moving, Prianto et al. [18] proposed to use collision detection (CD) to punish the robot, but the robot will not receive the penalty signal when touching the obstacle, which will lengthen the training time. Ota et al. [19] proposed a trajectory planning method for manipulators working in a constrained space to avoid obstacles outside the constrained space, but the definition of the constrained space has a certain particularity.

Inspired by the reference [20] on the recognition of the human lying position on the hospital bed, this paper further develops the research on the trajectory planning of the dual-arm robot to help patients turn over or to transport them from the bed. Considering the complicated situation between the human body and the environment, this paper proposes a DRL-based method to form the trajectory of both arms autonomously. In this study, when the dual-arm robot lifts a patient, the robot’s arms have to be inserted into the narrow space between the human body and the bed; they need to avoid obstacles to prevent the mechanical arm from colliding with the bed or the human body. Because the human body and the bed have complex shapes, this is relatively difficult to achieve. The major difficulty of this duty arises with the high dimension of the robot state and its behavior. When training the robot with DRL, the robot needs to control each joint of the robotic arm according to the position of the human body and the bed in the environment. The states and behaviors involved in this process are very complex to describe, which leads to slow convergence and long calculation time. This is also a classic problem in DRL—the curse of dimensionality. To tackle the problem of DRL, accelerate the training to find the optimal strategy, optimize the length of training step, and reduce the training time, this research designed the RPF based on the idea of the APF method. The RPF is composed of three parts: goal guidance, obstacle avoidance, and time function.

Due to the complex shapes of the human body and the bed, there are two problems setting the RPF during the reinforcement learning training process. One problem is that when the robot approaches the targets, with an alteration of the minimum distance between the end of the robotic arm and the targets, the robot may not obtain enough rewards to support the exploration of the environment. Therefore, a multifaceted consideration of the weight of the reward and the penalty signals during the training is needed. The other problem is that the collision constraint of the human body or the bed is complicated to represent. Multiple obstacle points and collision configurations have to be set up to construct the outline of the human body and the bed. Furthermore, the two problems will introduce a more challenging situation—while avoiding these obstacles contours the robot may also avoid the target areas between the human body and the bed. Therefore, it is also a challenging task to set the obstacle avoidance function (OAF) and CD reasonably to obtain desired results from reinforcement learning. In response to the above problems, this paper studies the dual-arm trajectory control strategies based on reinforcement learning. During the training process, based on the idea of the APF method, the RPF is designed, in which the reward guide function (RGF), OAF, CD, and time function are set up reasonably. According to the gravity function of the artificial potential field, RGF is designed based on the distance between the targets and manipulators, and it guides the robot to approach the targets continuously by updating the minimum distance between the end of the robot arm and the targets, and finally makes the end of the robot arm reach the required position. In terms of obstacle avoidance, inspired by the repulsion function and 13 obstacle points set to obtain penalty signals to achieve better performance, this paper combined it with the collision detection configuration. Compared with only using the CD method from [21], the present method further improves the efficiency of obstacle avoidance by reducing about 500 thousand training steps, and constructing a new RPF to ensure the agents receive more rewards. Furthermore, the time function is used to guide the agent to train faster. Finally, through simulation, the effectiveness of the RPF and obstacle avoidance method is verified, and the robot can accurately avoid obstacles to reach the expected targets. The contributions of this article are mainly the following three points:

(1) Proposed a dual-arm robot trajectory planning method based on DRL, which enables the robot to interact with the environment to find an optimal path to the targets;
(2) Designed the RPF based on the idea of the APF method, which creates a suitable signal for the robot to support the exploration of the environment and arrive at the ideal position to hold the patients;
(3) Combined OAF and CD, reducing the training time on obstacle avoidance and enhancing the stability of training.

**Figure 1:**
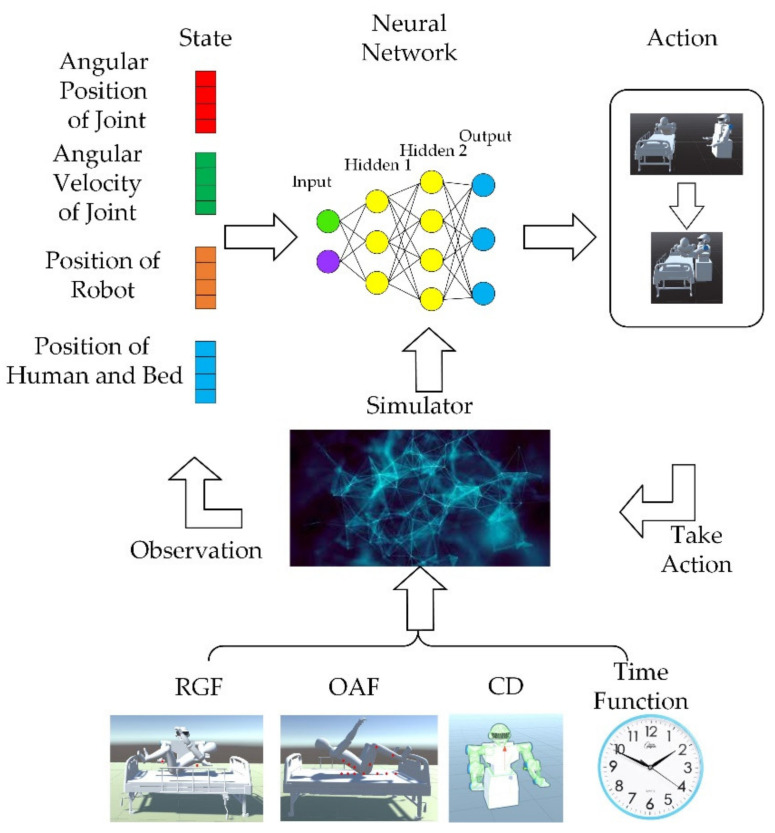
The graphical abstract of this article.

The rest of the article is structured as follows; Section 2 describes the model of the dual-arm robot and presents the preliminaries on deep reinforcement learning algorithms. In Section 3, according to the challenge mentioned above, the RPF setting method is proposed. The simulation result and discussion about the RPF setting are given in Section 4. Finally, conclusions are given in Section 5. The graphical abstract of this paper is shown in Figure 1 below.

## 2. Preliminaries on Robot and Deep Reinforcement Learning

### 2.1. Model of the Robot

**Figure 2:**
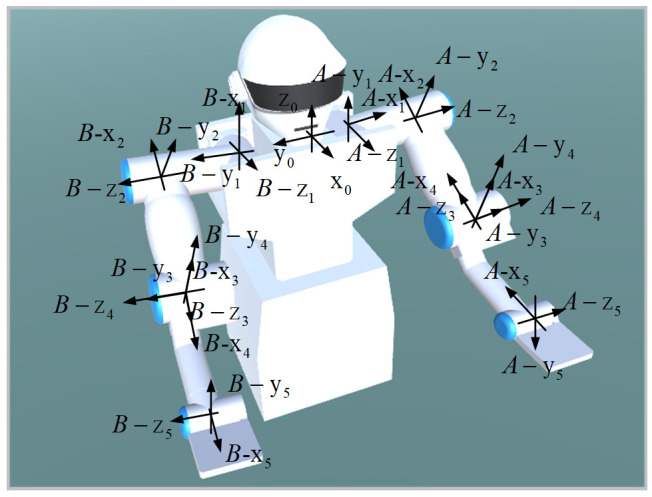
The overall structure of the robot.

The dual-arm robot studied in this paper has five rotating joints for every single arm. The left arm of the manipulator is marked as A, and the right arm is marked as B. The robot joint coordinate system can be established according to the Denavit–Hartenberg (DH) parameter method. As shown in Figure 2, $\{A-x_{i}(y_{i},z_{i})\}(i=1,2,3,4,5)$ and $\{B-x_{i}(y_{i},z_{i})\}(i=1,2,3,4,5)$, respectively, correspond to the 1~5 joints of both manipulators of the robot. The joints rotate with their *Z*-axis, respectively, as shown in Figure 2. The coordinates of the left and right arms are marked as $A_{e}$ and $B_{e}$. According to [22], the motion of both manipulators can be defined as Equation (1), the position and orientation vector of the end of the left arm can be defined as $P_{AR}$, and the right arm end position and orientation vector are expressed as $P_{BR}$ and $\Phi_{BR}$:

(1) $$X_{A}=\left[\begin{array}{c} P_{AR}\\ \Phi_{AR} \end{array}\right]\;X_{B}=\left[\begin{array}{c} P_{BR}\\ \Phi_{BR} \end{array}\right]$$

### 2.2. Deep Reinforcement Learning

**Figure 3:**
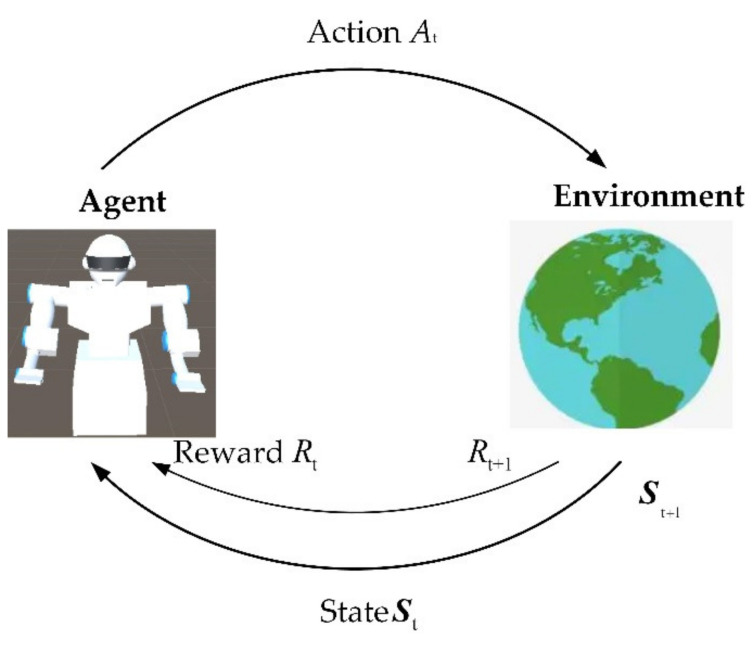
The agent–environment interaction in a Markov decision process.

DRL is a Markov decision process (MDP). The mathematical model of reinforcement learning is mainly composed of two parts, one is the agent, and the other is the environment, as shown in Figure 3.

MDP can be defined by a tuple $\left\{S,A_{c},P,R,\gamma \right\}$, where the state set of a limited environment $A_{c}$ is the set of actions performed by the agent in the environment, P is the state transition probability matrix, and γ is the discount factor, which is the future impact on the present. Furthermore, P is the immediate reward obtained by calculating the action after the environment receives action $A_{c}$ [23,24].

MDPs are meant to be a straightforward framing of the problem of learning from interaction to achieve a goal, and the process is shown as follows: The agent will interact with the environment and receives a state $S_{t}$ ($S_{t}\in S$) at the time step t in the sequence of discrete-time steps, and selects an action ($A_{t}\in A_{c}$). One step later, in part as a consequence of its action, the agent receives a numerical reward and finds itself in a new state $S_{t+1}$. The process thereby gives rise to a sequence that begins as [25].

The goal of reinforcement learning is required to find the optimal strategy that can obtain the largest long-term cumulative reward. The cumulative reward under strategy π is [26];

(2) $$R=R_{t+1}+\gamma R_{t+2}+\ldots =\sum_{k=0}^{\infty }\gamma^{k}R_{t+k+1}$$

DRL algorithms used in robot control mainly include DDPG, trust region policy optimization (TRPO), proximal policy optimization (PPO) algorithm, and other algorithms. Although the policy gradient algorithm has made some progress, the method is very sensitive to the number of iterative steps. If the number is too large, the feedback signal will be submerged in noise, and it may even lead to an avalanche of decline in model training. When the sampling efficiency is also very low, learning simple tasks requires millions of iterations. The PPO algorithm draws on the TRPO algorithm, uses first-order optimization, and achieves a new balance between sampling efficiency, algorithm performance, and the complexity of implementation and debugging. Therefore, this paper selects the PPO algorithm. The PPO algorithm is an improved algorithm based on the TRPO. After the conjugate gradient method is used to reduce the amount of calculation, the amount of calculation may still be large, which makes the algorithm not ideal in terms of speed. On this basis, the PPO algorithm improves the objective function so that the algorithm can use the gradient descent method to solve the model, which improves the simplicity of the algorithm and the efficiency of the solution.

In practice, the reinforcement learning algorithm uses the Monte Carlo method to approximate the expectation, and the objective function of the confidence region strategy for the optimization algorithm is shown in Equation (3) [27]:

(3) $$maximize\frac{1}{N}\sum_{t=1}^{N}[\frac{\pi (a|s)}{\pi_{old}(a|s)}A_{{}_{\pi old}}(s,a)]$$

where π(a|s) is the probability distribution on the new policy action set, $\pi_{old}(a|s)$ is the distribution of the old policy action set and is the estimator of the advantage function of the old policy.

The TRPO algorithm defines the trust region restriction strategy update for each round of optimization of the model, thereby ensuring the stability of the optimization. The trust region is limited by the Kullback–Leibler Divergence (KLD) in the constraint condition. It is required that the probability distribution between the new strategy and the old strategy is not too big. Therefore, the value of $r_{t}(\theta )$ can be considered to be close to 1. The PPO algorithm proposes another objective function, such as Equation (4) [28]:

(4) $$L_{t}^{CLIP}=\frac{1}{N}[\min(r_{t}(\theta )A_{\pi_{old}}(s_{t},a_{t}),clip(r_{t}(\theta ),1-\varepsilon ,1+\varepsilon )A_{\pi_{old}}(s_{t},a_{t}))]$$

where ε is the maximum difference between the new strategy and the old strategy, and $A_{\pi_{old}}(s_{t},a_{t})$ is the advantage function, which is the relative advantage of a certain action under the current strategy. To achieve the effect of the trust region in the TRPO algorithm, the algorithm has made two restrictions: The first part is the restriction on the probability ratio $A_{\pi_{old}}(s_{t},a_{t})$ of the new and old strategies. ε limits the probability ratio $A_{\pi_{old}}(s_{t},a_{t})$ of the new and old strategies to [1−ε,1+ε], which means, when $A_{\pi_{old}}(s_{t},a_{t})>0$, if $r_{t}(\theta )>1+\varepsilon$, then $L^{clip(\theta )}$ takes the upper limit $\left(1+\varepsilon )A_{\pi_{old}}(s_{t},a_{t}\right)$. When $A_{\pi_{old}}(s_{t},a_{t})<0$, if $r_{t}(\theta )<1-\varepsilon$, then $L^{clip(\theta )}$ takes the lower limit $\left(1-\varepsilon )A_{\pi_{old}}(s_{t},a_{t}\right)$, and it makes sure that each update does not have too much fluctuation. The second part is in the minimum function, which takes the lower value of the calculation as a result. The lower value can be optimized to the level of excellent performance, and then for other situations, the model will perform better.

At the same time, the PPO algorithm improves the value model, uses the generalized advantage estimator (GAE) algorithm to approximate the advantage function in the objective function, and adds the goal of the state value function and the entropy of the strategy model to the objective function. The complete objective function is shown in Equation (5) [29]:

(5) $$L_{t}^{CLIP+VF+S}(\theta )={\hat{E}}_{t}[L_{t}^{CLIP}(\theta )-c_{1}L_{t}^{VF}(\theta )+c_{2}S[\pi_{\theta }](s_{t})]$$

$L_{t}^{VF}={\left(V_{\theta }(s_{t})-V_{t}^{t\arg et}\right)}^{2}$ estimates the state value function, $S[\pi_{\theta }]$ of the strategy model is added to increase the diversity of the model, and the objective function can be solved by the gradient descent method.

## 3. Learning to Generate Motion

When training robots to generate a trajectory, it is time consuming and costs a lot to use real robots. Therefore, an effective simulation environment plays an increasingly important part in the application of robots. This paper chooses Unity as the reinforcement learning platform.

### 3.1. Introduction to Unity Real-Time Environment

**Figure 4:**
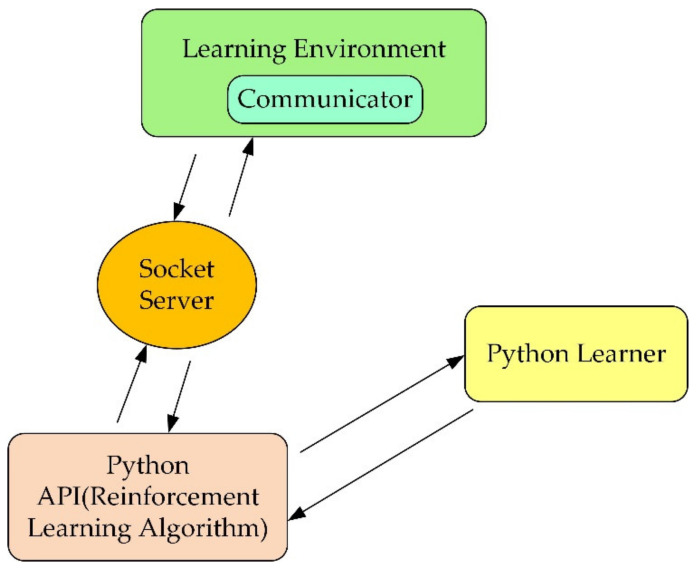
The relationship and structure diagram of the three major components of ML agents.

Unity is a real-time 3D interactive creation and operation platform. In this research, firstly, the robot 3D model needs to be imported into the Unity 3D environment. Secondly, before training the robot, it is necessary to use ML agents (machine learning agents) to establish the interactive communication between the simulation environment and the reinforcement learning and use the algorithm to train the robot. The operation mechanism of the environment is shown in Figure 4.

The learning environment is built with Unity, and the python application programming interface (API) contains machine learning algorithms. External communicator connects Unity with python API. When using ML agents, it is necessary to collect real-time information about the robot and determine the following action of the robot. Therefore, three parts must be defined at each moment in the environment:

(1) Observation is the robot’s view of the environment; the robot can collect environmental information through visual recognition.
(2) Action refers to the action the robot can take.
(3) A reward signal is a scalar value indicating how the robot behaves, which will be provided only when the robot performs well or badly rather than every moment during the training.

**Figure 5:**
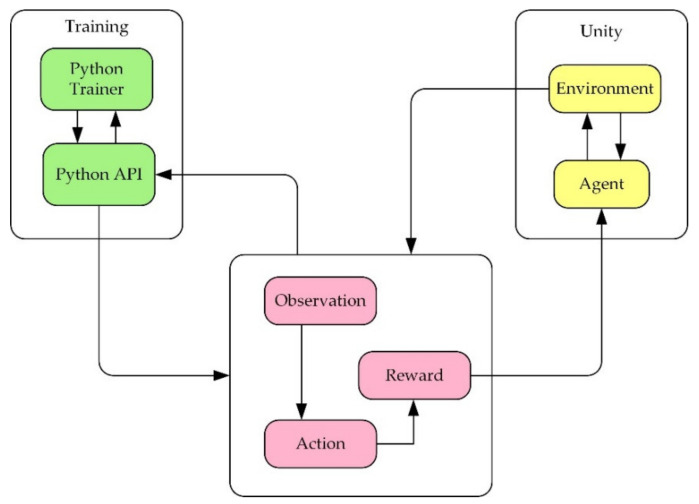
Training logic diagram.

After defining the above three parts, the train can commence. The logic of ML agents during reinforcement learning training is shown in Figure 5 below.

In training, regarding the robot as an agent, the agent can be used more than once, and the data obtained by the agents can be shared, which will accelerate the speed of training.

### 3.2. Action and State Initialization

**Figure 6:**
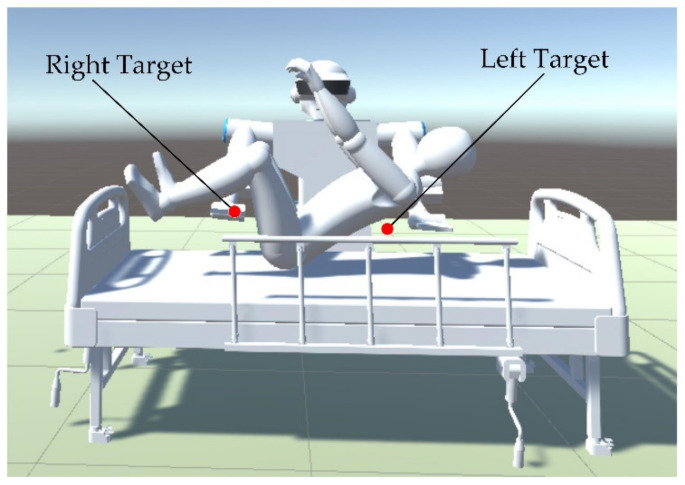
The target setting diagram.

Various entities in the environment need to be properly set for better training. The trajectory that the robot’s arms need to generate should consider the position of the robot and other obstacles. According to the operating range and reasonable service area of the robot, the targets are supposed to be under the back and knees of the man. The position of the obstacle (bed, pillow, and the human body) remain unchanged. To ensure the stability of training, the positions of the targets, the robot, and the obstacle should avoid conflicts. In the experimental environment, to achieve effective training of dual-arm robot trajectory planning, it is necessary to observe the position of the robot, the targets, and robot joints. With the information that can be obtained in the actual service environment, the joint rotation of the dual-arm robot, the coordinates of the targets, and the coordinates of the robot, here the position observation set $O_{p}$ is defined as:

(6) $$O_{p}=(P_{AJ_{i}},P_{BJ_{i}},P_{G},P_{R})(i=1,\;2,\;3,\;4,\;5)$$

where $P_{AJ},P_{BJ}$ is the position of the left and right manipulators, respectively. $P_{G}$ and $P_{R}$ are the position of targets and the obstacles, respectively. Whether the human body can be lifted successfully is decided by the position of the targets and the posture of the human body during lifting. There are five joints for each robot arm of the dual-arm robot. Trajectory planning for the dual-arm robot aims to find the shortest and collision-free trajectory to reach the targets and posture after setting the initial information. The targets of the arms are considered to be under the shoulders and the knees of the human body for the convenience of holding. The center of the targets area is shown by the red dot in Figure 6. According to the model in Section 2.1, the initial and targets configuration of the robot can be expressed as follows:

(7) $$X_{inti}=\left[\begin{array}{cc} P_{ARinti} & P_{BRinti}\\ \Phi_{ARinti} & \Phi_{BRinti} \end{array}\right]\;\;X_{goal}=\left[\begin{array}{cc} P_{ARgoal} & P_{BRgoal}\\ \Phi_{ARgoal} & \Phi_{BRgoal} \end{array}\right]$$

where $X_{inti}$ represents the initial configuration including position and orientation of the robot, $X_{goal}$ represents the targets configuration of the robot, $P_{ARinti}$ represents the initial position of the left manipulator, $\Phi_{ARinti}$ represents the initial posture of the end of the left manipulator, other symbols are similar. The motion control drives each joint angle so that the end of the robotic arm can reach the targets. Here, by limiting the range of joint angle changes during the training process, the robotic arm will explore the training environment constantly, and feed back the value of rewards. We let the joint space of the robot be Q, and it can be expressed as follows:

(8) $$Q=\left[\begin{array}{ll} q_{A1} & q_{B1}\\ q_{A2} & q_{B2}\\ q_{A3} & q_{B3}\\ q_{A4} & q_{B4}\\ q_{A5} & q_{B5} \end{array}\right]$$

The exploration of the robot’s dual-arm joint angle is as follows:

(9) $$\begin{array}{l} q_{Aj}=q_{A0j}+15ContinuousAction[j]\\ q_{Bj}=q_{B0j}+15ContinuousAction[j] \end{array}$$

where $q_{Aj}$ and $q_{Bj}(j=1,2,\ldots ,5)$ represent the joint angles of the left and right arms of the robot, $q_{A0j}$ and $q_{B0j}(j=1,2,\ldots ,5)$ is the initial value of each joint angle, and 15ContinuousAction[j] means that the output of the joint, which is calculated by reinforcement learning and its range of change, is limited to [−15,15].

### 3.3. Training Scheme

After the environment is established, the training agent scheme can be further determined.

In this paper, we considered training multiple agents with a single brain, every agent associated with a robot. That means there are multiple independent reward signals and multiple independent agents communicating with each other, collecting data, and calculating gradients. The data is summarized together to update network parameters, and mutual feedback between strategies is carried out.

**Figure 7:**
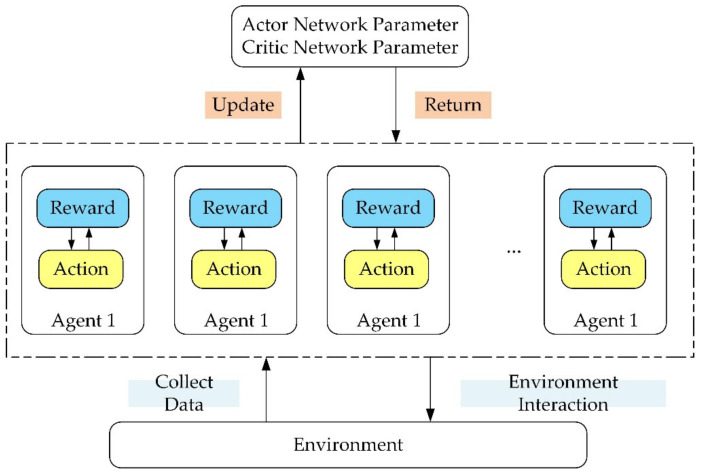
Training scheme.

Figure 7 shows that multiple agents are working at the same time during the training. Every agent is independent, but the data obtained will be uploaded to the same network for parameters updating, and the reward signals will be feedback to the agent as evidence for taking action. With repetition, the training will finally achieve the goal.

### 3.4. The Reward Guidance Mechanism Design

After initialization of the action state of the robot, the agent can randomly derive different action strategies according to the state, but it cannot evaluate the quality of the action according to the state. The design of the reward guiding the function will evaluate the behavior of the agent and increase the probability of high-scoring behavior. The reward mechanism determines the effect of the training results. A reasonable RPF will increase the training speed, reduce the consumption of computer resources, and make the training converge faster. In most cases, continuous reward and punishment information can continuously allow the agent to get feedback on the action strategy adopted, which is more effective than sparse reward signals.

This paper is inspired by the idea of the APF method and sets up a continuous reward function. There are three main considerations in setting up the reward mechanism: (1) reaching the targets; (2) avoiding obstacles; (3) minimizing training time. For problem (1), this paper sets up a guidance function, which will reward the robot when it approaches the targets and punish it when it moves away. For problem (2), two methods are proposed here. One is the obstacle avoidance function (OAF), inspired by the idea of the APF method. The second is collision detection (CD) which will punish the agent if the robot collides with obstacles. The OAF will penalize the robot when it approaches obstacles; the closer the distance, the higher the penalty. As the penalty signal is continuous, the training time will be shorter compared with CD, but whether the selection of obstacle points is representative will affect the training. The agent will be unable to identify obstacles if the obstacle points do not describe the obstacle well. The signals obtained by CD will penalize the robot only when the robot collides with the obstacle, which means the reward signal is discrete. The training time will be longer compared with OAF, but because the CD will divide the entire workspace into two parts, $W_{collide}$ and $W_{free}$, the CD will have a more comprehensive description of obstacles, and the training effect will be more stable. The two methods have their advantages and disadvantages. In the specific implementation process, it is necessary to set the weight of the penalty of the two methods reasonably to obtain better results. For the problem (3), a time function is set, and a constant penalty is given for each round of training to reduce the training time.

#### 3.4.1. Reward Guide Function

The guide function setting is inspired by the traditional APF method when setting the reward function for path planning. The gravity of the APF method is determined by the current position of the object, the gravity is represented by the reward signal, and the reward signal is determined by the action of the agent. Otherwise, the agent will be punished. For example, in a certain state $S_{t}$, there is a certain distance between the end $P_{Aend}=(x_{At},y_{At},z_{At})$, $P_{Bend}=(x_{Bt},y_{Bt},z_{Bt})$ of the dual-arm robot, and the targets $P_{Agoal}=(x_{Ag},y_{Ag},z_{Ag})$ and $P_{Bgoal}=(x_{Bg},y_{Bg},z_{Bg})$, which is represented by the distances $dis_{At}$ and $dis_{Bt}$. If the distance is continuously reducing, it means that the robot’s action strategy is correct, and the behavior should be rewarded. The guide function equation is set as:

(10) $$\begin{array}{l} dis_{At}=\sqrt{{\left(x_{At}-x_{Ag}\right)}^{2}+{\left(y_{At}-y_{Ag}\right)}^{2}+{\left(z_{At}-z_{Ag}\right)}^{2}}\\ dis_{Bt}=\sqrt{{\left(x_{Bt}-x_{Bg}\right)}^{2}+{\left(y_{Bt}-y_{Bg}\right)}^{2}+{\left(z_{Bt}-z_{Bg}\right)}^{2}} \end{array}$$

(11) $$dis_{Amin/Bmin}=\left\{\begin{array}{cc} \;dis_{0} & t=0\\ \min(dis_{At/Bt},dis_{Amin/Bmin}) & t>0 \end{array}\right.$$

(12) $$R_{g}=\left\{\begin{array}{cc} k_{1}(dis_{Amin/Bmin}-dis_{At/Bt}) & \left(if\;dis_{Amin/Bmin}<dis_{At/Bt}\right)\\ k_{2}(dis_{Amin/Bmin}-dis_{At/Bt}) & \left(if\;dis_{Amin/Bmin}>dis_{At/Bt}\right)\\ \;k_{3} & \left(if\;dis_{Amin/Bmin}=0\right) \end{array}\right.$$

In the Equations (10)–(12), $k_{1}$, $k_{2}$, and $k_{3}$ are appropriately selected positive constants, $dis_{At}/dis_{Bt}$ represents the distance between the end of the robot’s arms and the targets at time t, and $dis_{Amin/Bmin}$ represents the minimum distance between the end of the robot’s arms and the targets at time t. They are not constants and will change with time. Equation (12) gives the update method: ($x_{At}$, $y_{At}$, $z_{At}$) and ($x_{Bt}$, $y_{Bt}$, $z_{Bt}$), respectively, represent the position coordinates of the end of the left and right arms of the robot at time *t*. The meaning of RPF is to take the minimum distance $dis_{Amin/Bmin}$ between the end of the robot arms and the targets at time *t*. When the time changes if the newly generated distance $dis_{At}/dis_{Bt}$ is less than $dis_{Amin/Bmin}$, it means that the distance between the end of the robot’s arms and the targets is decreasing. Then a reward $k_{2}(dis_{Amin/Bmin}-dis_{At/Bt})$ can be given. If $dis_{At}/dis_{Bt}$ is greater than $dis_{Amin/Bmin}$, it means that the distance between the end of the robot’s arms and the targets is increasing, and a penalty $k_{1}(dis_{Amin/Bmin}-dis_{At/Bt})$ is given. When the distance $dis_{At}/dis_{Bt}$ is zero, it means that the robot has reached the targets correctly. The training is deemed successful, and the highest reward $k_{3}$ is given, and then this round of training can be ended. However, when the end of the robot’s arms gets closer and closer to the targets, the reward will be lowered, which will affect the training speed. Therefore, $k_{1}<k_{2}$ is set here, that means the weight of the reward is greater than the weight of the penalty.

#### 3.4.2. Collision Detection

This part mainly focuses on how to avoid the obstacle. Here are two main ideas for obstacle avoidance. One is to set up collision detection (CD), which is to set penalties by detecting whether the robotic arm collides with obstacles, and the other is to set obstacle avoidance function (OAF), inspired by the idea of the APF method. The robot will obtain different penalty signals according to the distance between the end of the manipulator and the obstacle. The OAF will improve the training faster than the CD, according to what is mentioned before, but the selection of obstacle points cannot represent all the shapes of the human body and the bed after all; therefore, to construct a better RPF, the CD and the OAF should complement each other. This section will first introduce CD.

In real life, collision detection is realized using an impact sensor, but in the virtual environment in Unity, it is realized in other ways. Let the operating space of the robotic arm be W, which can be divided into two subsets, $W_{collide}$ and $W_{free}$, the former represents the space where the obstacle stays, the latter represents the space where the robot can move freely, and the space occupied by the robot is M. If the robot belongs to $W_{free}$, it means the robot does not collide with any obstacles. If the robot arm belongs to $W_{collide}$, it means that there is a collision between the obstacle and the robot arm. The division of $W_{collide}$ and $W_{free}$ will be taken by CD. The CD is mainly about the collision between the robot arm and the obstacle. The obstacle here specifically includes the human body and bed.

**Figure 8:**
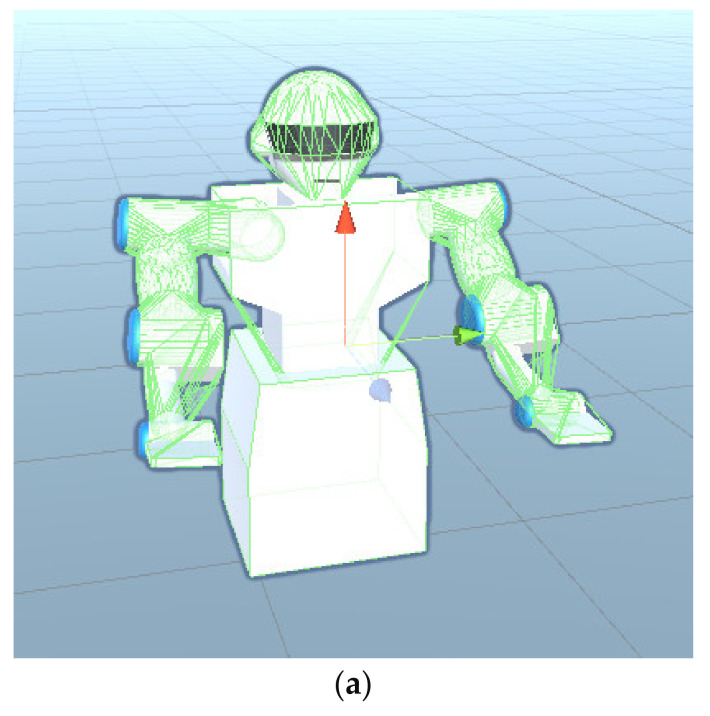

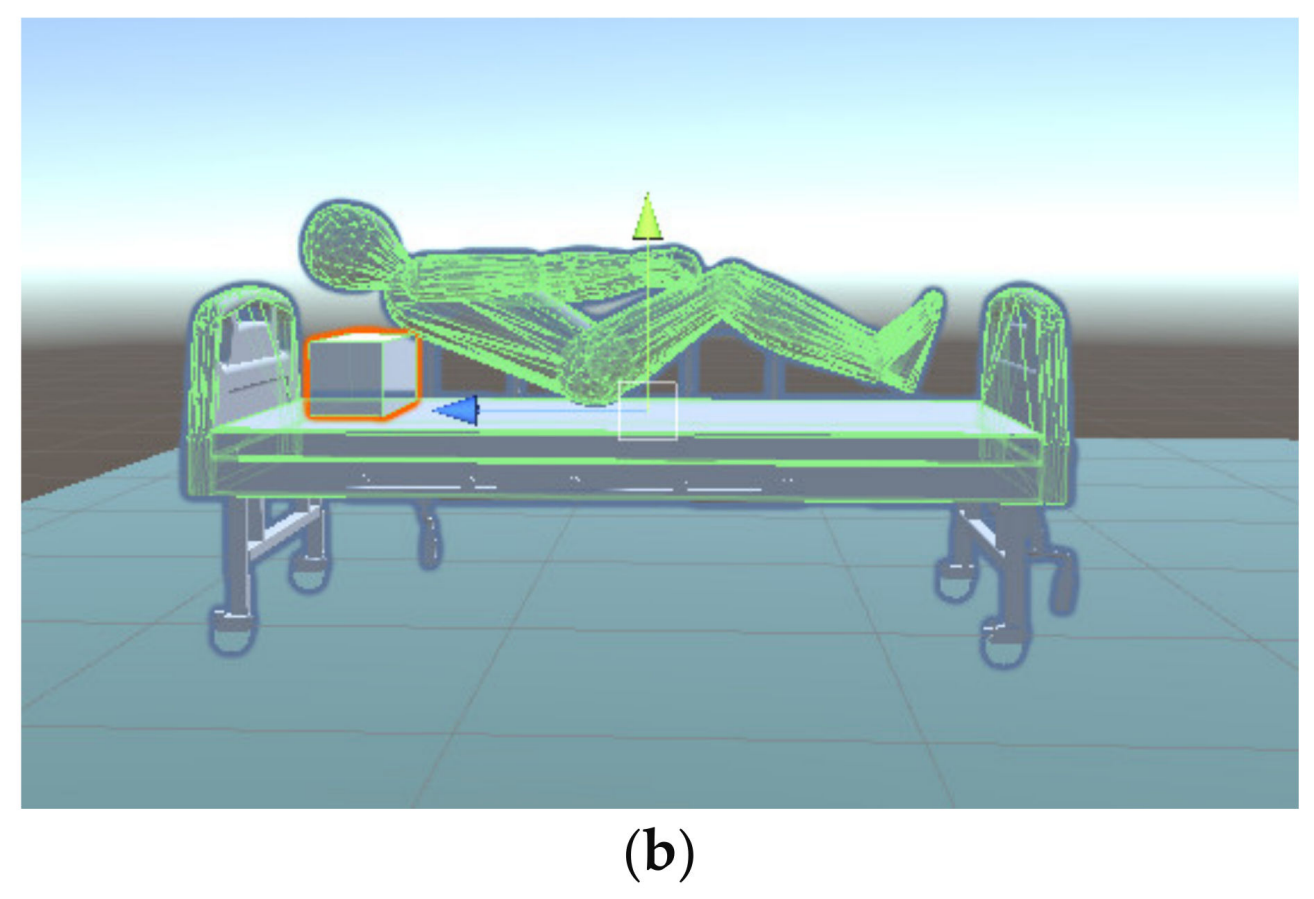
Collision detection configuration diagram. (**a**) Robot collision detection configuration. (**b**) Human body and bed collision detection configuration diagram.

The CD is used to detect whether objects collide with each other [18]. If the object is very regular, such as a sphere, it is easy to directly detect whether the distance from the center of the circle is less than the radius. However, if the object is irregular, such as a robot, it will become very difficult to distinguish whether two objects collide with each other. Therefore, it is necessary to use simple geometry to approximate complex shapes of objects. Taking the situation mentioned above into consideration to obtain more accurate training results, the human body and robot arms are shaped by colliders, which will cover a layer of the grid on the surface of the robot, the human body, and the bed. During the detection, the collider will check whether the robot manipulators collide with obstacles. The configuration of the collision detection between the robot and the human body is shown in Figure 8:

According to collision detection, the penalty function can be set as follows:

(13) $$R_{o}=-k_{o}\;(\mathrm{if}\;M\cap W_{collide}\neq \emptyset )$$

where $k_{o}$ is a positive constant and $M\cap W_{collide}$ means that the robot collides with the obstacles; if there is a collision, the agent will be punished with a penalty $-k_{o}$.

#### 3.4.3. Obstacle Avoidance Function

The APF method makes the robot avoids obstacles by setting the repulsive force function. The repulsive force function is designed according to the distance between the robot and the obstacle. Here the idea of the function to design the penalty function is considered, taking the reciprocal of the distance between the end of the robot manipulators and the targets as the penalty signal. In the real world, to recognize the shape of an object, there are always some mark points attached to it. In this subsection, an arrangement method of the mark points is proposed.

**Figure 9:**
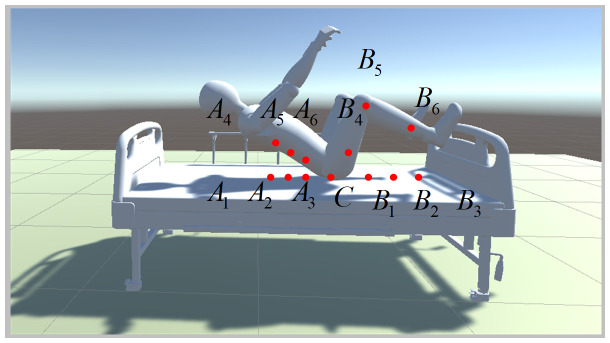
Diagram of Barrier point.

Both the human body and the bed have the characteristics of complex shapes. The OAF designed according to the APF method is like the repulsive force between points, and it is necessary to calibrate the shape of the human body and the bed, as shown in Figure 9.

According to Figure 9, it is known that the targets of the robot are below the back and the knees of the human body. The obstacle points $A_{i},B_{i},C$ (*i* = 1, 2, …, 6) in Figure 9 are set to ensure that the robot will not collide with the human body or the bed while approaching the targets, where the area enclosed by points $A_{i}$ and C is the ideal activity space for the left arm, and the area enclosed by points $B_{i}$ and C is the ideal activity space for the right arm. It can be seen that point C divides the movement space of the left and right arms to prevent the left and right arms from being too close during work. This arrangement has two advantages. One is to prevent collisions between the left and right arms of the robot, and the other is to enable the robot to use a proper posture to pick up people.

After setting the obstacle point, we can set the OAF, as shown below:

(14) $$R_{o}^{\prime }=-k_{o}^{\prime }(1/dis_{Ai}+1/dis_{AC}+1/dis_{Bi}+1/dis_{BC})(i=1,2,\ldots ,6)$$

where $k_{o}^{\prime }$ is a positive constant, $dis_{Ai}$ represents the distance between the end of the left arm of the robot and $A_{i}$, $dis_{AC}$ represents the distance between the end of the left arm of the robot and point *C*, $dis_{Bi}$ represents the distance between the end of the right arm of the robot and point $B_{i}$, and $dis_{BC}$ is the distance between the end of the right arm of the robot and point *C*. As can be seen from Equation (14), when the end of the robot is far away from the targets, that is, when the $dis_{*}(*=Ai,AC,Bi,BC)$ is large, the penalty obtained by the robot, namely, $1/dis_{*}$ can be ignored. When the distance is too close, the penalty is $1/dis_{*}$ will tend to infinity. This produces a continuous penalty signal for the robot, and at the same time, it can also obtain the penalty signals when the robot is approaching targets compared with the CD, which will speed up the training.

#### 3.4.4. Time Function 

In the simulation training, to reduce the training time, a constant penalty item can be set, and a penalty will be performed for every additional round of training. The time penalty function is represented by $R_{p}$, which can be set according to the length of the training time, but the value should not be set too large; otherwise, the robot will be unable to perform effective exploration because of the excessive punishment in the early stage of training. The function equation can be expressed as:

(15) $$R_{p}=-k_{t}$$

where $k_{t}$ is the time penalty constant, which is positive.

The design of the total reward function is the cumulative sum of the above three functions, which can be expressed as:

(16) $$R=R_{g}+R_{o}+R_{o}^{\prime }+R_{t}$$

### 3.5. Training Process

The goal of reinforcement learning is to find the optimal strategy for maximizing the total rewards of the agent in the path planning. If the iterative training time is limited, the agent can find the trajectory to the goal by maximizing the total rewards, but when the agent does not receive enough training, it is difficult to find the targets because the action is mainly determined by experimental methods, which means that many iterations cannot reach the targets before the agent is well trained. In this case, there are only a few states and actions helpful for learning. The process is called the Markov process with sparse reward. To overcome this problem, this paper designed a trajectory planning algorithm using the PPO algorithm. The PPO algorithm is known for its easy implementation and high efficiency. It improves the sample efficiency of the scant reward in the DRL training.

According to Section 2.1, the state S could be expressed as:

(17) $$S=\{p_{A},n_{A},p_{B},n_{B}\}$$

where $p_{i}$ and $n_{i}(i=A,B)$ represent the positions and orientations of the end of the manipulator $p_{i}$ and $n_{i}(i=A,B)\in W_{free}$, which means that state quantities are considered to belong to free space.

The action $A_{c}$ can be defined by Equation (9), where $q_{A}$ and $q_{B}$ represent the angle vector of each joint of both arms of the robot. The action at time t means that the robot must meet the joint position at time *t* + 1:

(18) $$A_{c}=\{q_{A},q_{B}\}$$

**Figure 10:**
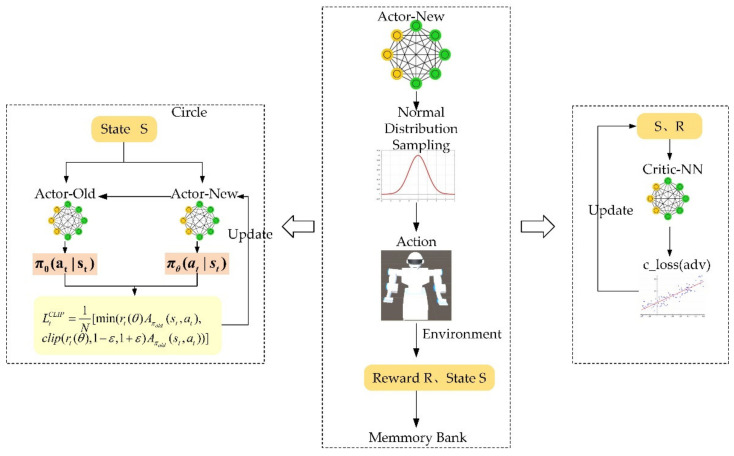
PPO algorithm network training flowchart.

The PPO algorithm needs to build three neural networks: Actor-New—the new policy network, Actor-Old—the old policy network, and Critic-nn—the evaluation network. The old and new strategic network is proposed to predict the action strategy, and the evaluation network is responsible for evaluating the effect of the action. During the training, firstly, the environmental observation information $O_{p}$ is input to the new strategic network. The new strategy network obtains the normal distribution parameters according to the observed environmental information, and action is sampled. The action interacts with the environment to generate rewards or punishments and obtain the next state $S_{t+1}$. After storing the state, action, rewards, and punishments in the memory bank, the state $S_{t+1}$ is then input into the new strategic network. This process repeats continuously until the storage capacity in the memory bank meets the requirements. In the evaluation network, through the continuous acquisition of observations and rewards, the agent will perform a back-propagation update of network parameters so that the evaluation value of the evaluation network for different situations is getting closer and closer to the setting value of the reward function; at the same time, the old and new networks output strategies according to the state set, and calculate the weights and update the parameters of the new strategic network according to Equation (17). After training for a certain time, the agent will use the new strategy network parameters to update the old strategy network parameters. This process repeats continuously until the set number of training steps is reached, and the training is completed. The training process is shown in Figure 10. The PPO algorithm is based on the actor–critic framework but with the style of policy gradient at the same time. In a specific implementation, when the Actor-New network obtains the environment information ***S***, it will obtain two values. Using these two values, a normal distribution can be constructed. The action will be sampled from this normal distribution. The obtained action interacts with the environment to obtain the reward ***R*** and state ***S*** of the next step. Reward ***R*** and state ***S*** will be stored in a memory bank, and the ***R*** and ***S*** in the memory bank will be input into Actor-New, and so on. The critic network calculates the reward value according to the obtained information and updates the network according to c_loss (adv). The actor network update method is similar to the critic network.

## 4. Simulation Result Analysis

The experimental platform is configured in Windows 10 system equipped with NVIDIA GeForce GT 710 graphics card and i5-6400 processor of Dell. To verify the effectiveness of the system, the analysis of the training result is conducted, as shown below.

### 4.1. Training Environment

**Figure 11:**
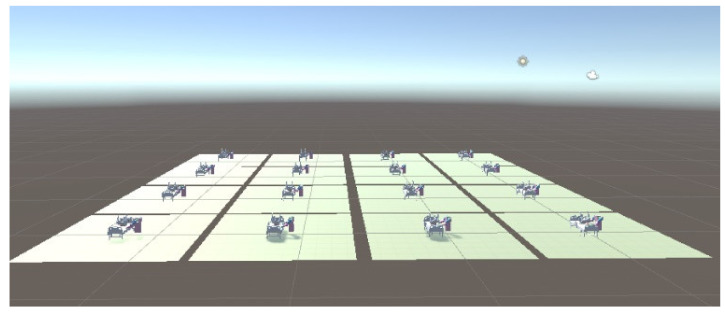
Training environment.

In the experiment, there are 16 agents, and the training environment adopts a lightweight layout, as shown in Figure 11.

After the simulation environment is configured in Unity, the PPO algorithm will be called from the Python API to train the robot. The relevant parameters of the algorithm are shown in Table 1. The maximum number of training steps is set to 500 million (of course, it can be stopped in advance when the desired results are obtained), the entropy regularization strength β (Beta) is set to 0.001, the acceptable difference range value ε (Epsilon) of the new and old strategies is set to 0.2, the number of hidden layers of the network is 128, and the reward signal parameter is set to 0.99. 

**Figure 12:**
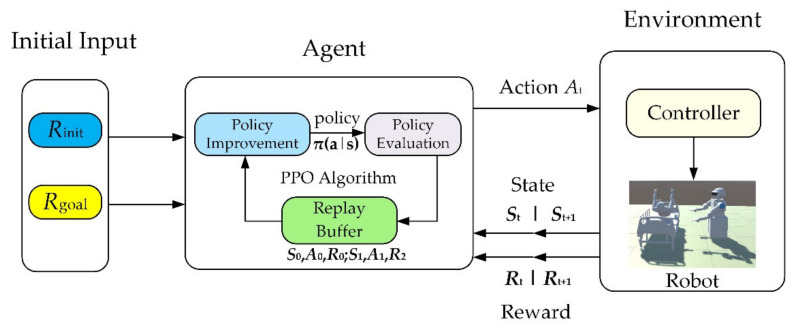
The Agent training process of generating trajectory.

**Figure 13:**
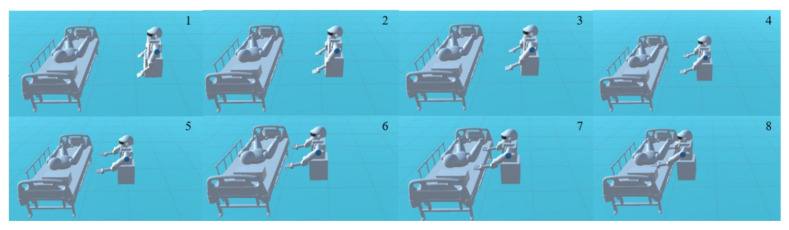
Diagram of training results. The number in the upper right corner of the picture represents the moving order of the robot.

In the training process, $\left(R_{\mathrm{int}i},R_{goal}\right)$ is input into the agent at the beginning, and the action will be output. The reward and state are generated at the same time and will be returned to the agent later. The same process will repeat until the expected result is achieved. Figure 12 shows the process of path formation:

After the agent is trained well, it will calculate the shortest path from the initial point to the targets according to the RPF. The training result is shown in Figure 13, where the human body is placed at a certain position on the left, and the robot is in a certain space on the right. The robot will move randomly to explore the environment to reach the targets for lifting the human body. It can be seen that the robot finally reaches the targets without collisions with the bed and the human body.

### 4.2. Training Analysis

To verify the effectiveness of the method in this paper, the robot performance under different RPF is carried out. The simulation in this paper is as follows: (1) No penalty function, that is, in the process of approaching the targets, if the robot collides with an obstacle, only the number of collisions is recorded, which means there is no penalty. (2) The RPF based on CD is used to detect whether the robot arm collides with the human body or the bed, and only if a collision occurs will the agent be punished. (3) The RPF is based on the OAF, and the advantage of the OAF is that the rewards or punishments the agent receives are continuous when approaching the targets. (4) RPF with OAF and CD, namely the combination of (2) and (3). Among all the RPFs mentioned above, the targets guidance mechanisms are all the same. Table 2 shows the training effect of all the RPFs. The times of success are the times the robot avoids the obstacle successfully to reach the expected position. The usage time is the time it takes to repeat the action 200 times. It can be seen from Table 2 that the success rate of training without penalty is only 20%, and the result is very bad. Because the space for the robot to approach the targets is relatively narrow, the robot arms can easily touch the bed or the human body, which is not allowed. The test results reflect the importance of obstacle avoidance for training.

**Figure 14:**
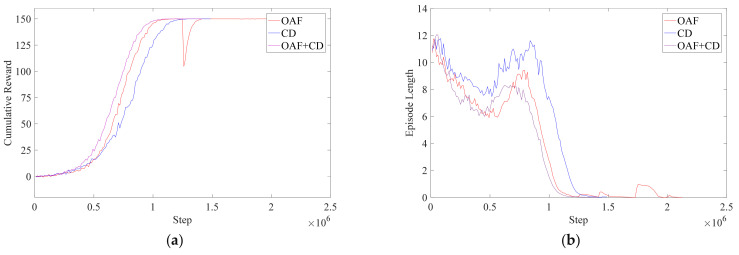
Training results diagram. (**a**) Cumulative Reward. (**b**) Episode Length.

The data during the training can be obtained through a tensorboard-1.7.0, and the data downloaded are imported into Matlab 2019b for drawing, as shown in Figure 14. The results obtained after training are as follows: It can be seen from Figure 14a that, compared with only one method of OAF or CD, the convergence of the combination of the two methods is the fastest. The use of both methods reduces the time by about 500,000 steps compared with the OAF and reduces about 1 million steps compared with the CD. The reward curve does not change sharply, which reflects the stability of the training results. Furthermore, it can be seen in Figure 14b that in terms of the curve of the episode length, the training result in this section is generally at the lowest position, and the time to zero is also the shortest.

From Figure 14, on the problem of obstacle avoidance, the CD can achieve a complete division of the workspace and a more comprehensive description of obstacles and robot shapes; hence the curve of the training results seems more stable because the penalty signal is more specific. However, since the CD gives the agent a penalty signal only when the end of the robot arm collides with an obstacle, the training time of the CD is longer. The use of the OAF requires the preselection of obstacle points, and then the agent will receive a penalty signal according to the distance between the robot arm and the obstacle point. The farther the distance is, the smaller the penalty is, and the closer the distance is, the greater the penalty. Therefore, the OAF is more sensitive to obstacles than the CD and will make the penalty signal continuous, which will effectively reduce the training time required for obstacle avoidance. However, due to the complex shapes of the human body and the bed, whether the selected obstacle points are representative will decide whether the robot can effectively avoid obstacles. According to the advantages and disadvantages of the CD and the OAF, this paper combined the two methods to construct a new RPF. Furthermore, the RPF has been proved to be faster and more effective than the OAF or the CD, according to the data analysis above. 

**Figure 15:**
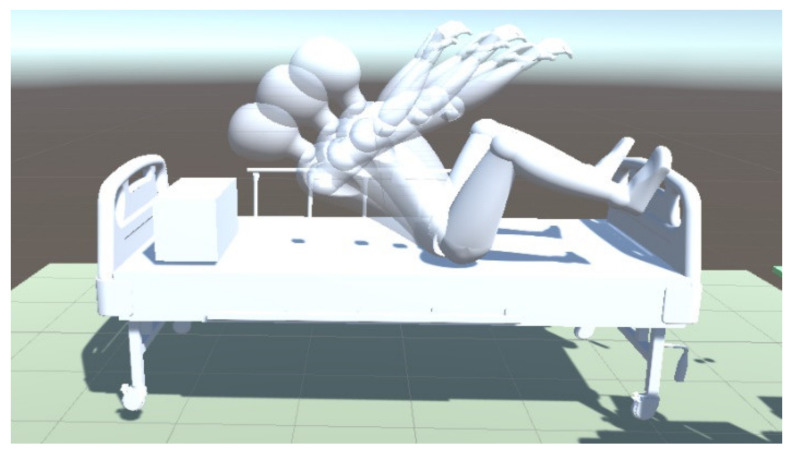
The human posture changing diagram.

**Figure 16:**
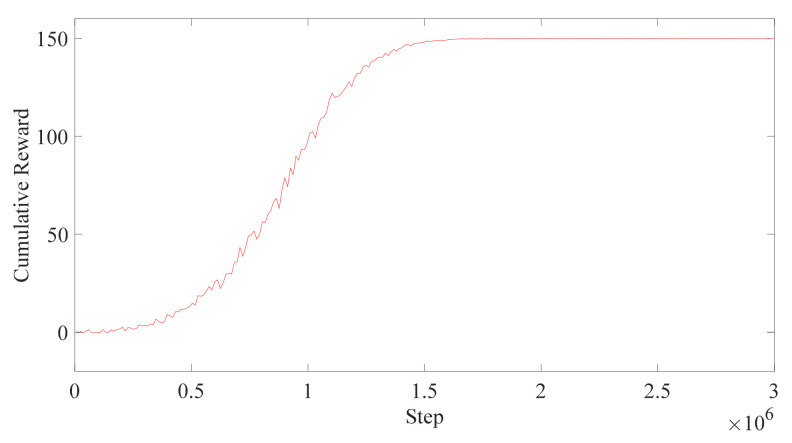
The training results in posture changing.

To show the advantage of the reinforcement learning and PPO algorithm further, another experiment is conducted. When the robot is approaching the targets, the posture of the human changes within a certain range when training, and the DDPG algorithm is used to accomplish the task again. The posture change diagram and rewards curves of both training are shown in Figure 15 and Figure 16.

**Figure 17:**
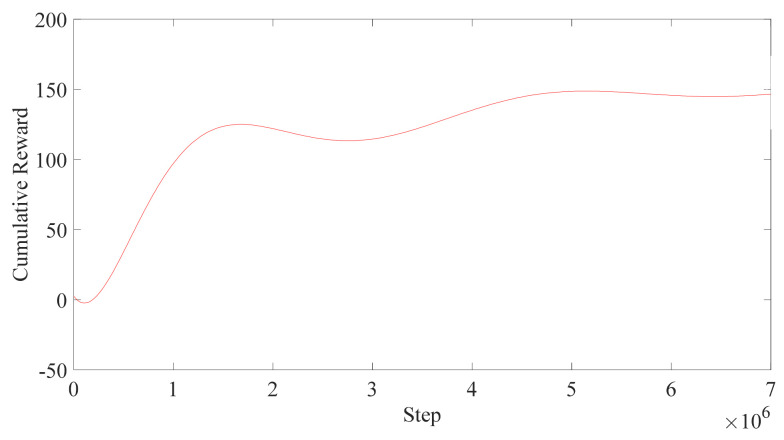
The training result of DDPG.

From the training result in Figure 16, it can be seen that even though the human posture is changing, the trajectory can still be generated, which shows the advantage of the PPO algorithm. However, there are another 500 thousand steps for training, which means that the task is becoming harder. From Figure 17, it can be seen that it takes many more steps for the robot to obtain the same results; where the DDPG algorithm takes 5 million steps, the PPO only takes 1 million steps. The rewards curve of the DDPG is fluctuant, which means that the algorithm is not that good for the robot to generate the trajectory, which shows the advantage of the PPO algorithm again.

## 5. Discussion and Conclusions

The research is conducted to provide inspiration for the application of robots in the field of medical care and reduce the social burden.

This paper carried out the trajectory planning research with the reinforcement learning of the dual-arm robot on the assistance with the patient and designed a kind of reward function which consists of RGF, CD, OAF, and time function. Where the RGF is used to guide the robot to reach the targets, the CD and OAF are used to avoid obstacles, and the training time is reduced, enhancing the stability of the training effect by using the RPF. The time function is used to make the agent train faster. The RPF effectively enables the robot to obtain a higher reward and alleviate the negative effect of the sparse reward problem of robot training in a high-dimensional environment.

As for the problem of obstacle avoidance, this article explored the use of the CD and the OAF, the two methods complementary to each other, considering both advantages and disadvantages of both, and the training results showed their superiority. Finally, to show the superiority of the PPO algorithm further, the DDPG algorithm is applied for the research, and the latter shows slower training and fewer rewards for the robot when accomplishing the task.

However, the robot only accomplishes the trajectory planning for holding the human up as a preparatory action, and the force information has not been considered, which may hurt humans during the operation. Furthermore, to study the problem more deeply, next, we will use force/position control to complete the task with deep reinforcement learning algorithms.

## Figures and Tables

**Table 1:** Hyper-parameters and reward signals.

Name	Value	Name	Value
Batch size	64	γ	0.99
Buffer size	12,000	Strength	1.0
Learning rate	0.0003	Keep checkpoints	5
β	0.001	Max steps	500,000,000
ε	0.2		
Lambda	0.99	Time horizon	1000
Number epoch	3	Summary frequency	12,000
Hidden units	128	Number layers	2

**Table 2:** The effect of different RPF.

Method	RPF Category	Times of Success (Total Number of Experiments)	Success Rate/%	Usage Time/s
1	Accessibility penalty function	40 (200)	20	124
2	RPF based on CD	183 (200)	91.5	152
3	RPF based on OAF	191 (200)	95.5	133
4	RPF based on CD and OAF	198 (200)	99	128
